# Potential head injuries in men's football, futsal and beach soccer: Distinct exposure‐adjusted frequency and patterns from a comparative video analysis

**DOI:** 10.1002/ksa.70158

**Published:** 2025-10-27

**Authors:** Yavuz Lima, Levend Karaçoban, Andreas Serner, Kerry Peek, Ogün Köyağasıoğlu

**Affiliations:** ^1^ Department of Sports Medicine, İstanbul University‐Cerrahpasa Cerrahpasa Faculty of Medicine İstanbul Turkey; ^2^ Department of Sports Medicine, Ankara University Faculty of Medicine Ankara Turkey; ^3^ FIFA Medical, Fédération Internationale de Football Association Zurich Switzerland; ^4^ School of Health Sciences, Faculty of Medicine and Health The University of Sydney Australia

**Keywords:** ball‐related impact, incidence, overhead kick, possible concussion, sports medicine

## Abstract

**Purpose:**

Football, futsal and beach soccer differ in playing conditions, but data on differences in head injury characteristics are limited. The aim of this study was to systematically analyse and compare potential head injuries in these disciplines.

**Methods:**

Footage from 148 matches across three men's international tournaments (2022 FIFA World Cup, 2024 FIFA Futsal World Cup and 2024 FIFA Beach Soccer World Cup) were reviewed to identify potential head injuries (any event where a player remained down >5 s and/or requested medical‐attention with the body part involving head/neck), player actions, visible signs of possible concussion, medical‐assessments and outcomes. Incidence rates (IRs; number of potential head injuries per 1000 match‐hours) were reported separately for each discipline based on official and total exposure times (the duration from initial to final whistle).

**Results:**

A total of 395 potential head injuries were identified: 186 (IR:85.8/1000 match‐hours) in football, 86 (IR:247.8) in futsal and 123 (IR:627.5) in beach soccer. After exposure adjustment, IRs were 76.4, 122.3 and 383.2, respectively. Direct opponent contact was the main mechanism for all disciplines (football 81.5%, futsal 76.2%, beach soccer 78.9%). Unintentional ball‐to‐head impacts accounted for 6% of cases (football), 16.7% (futsal), and 13.8% (beach soccer). In beach soccer, 17.9% of injuries were associated with overhead kicks. Visible signs of a possible concussion were observed in 16.3% of cases (football), 9.5% (futsal) and 20.3% (beach soccer).

**Conclusion:**

Incidence and patterns of potential head injuries differ across football, futsal and beach soccer, underscoring the need for individual recognition in all three disciplines. Ball‐related impacts contributed significantly to the differences in IRs and overhead kicks posing a unique risk in beach soccer. Targeted preventive strategies, such as specific drills on safe blocking in futsal, and training to improve the safe execution of overhead kicks in beach soccer, may help reduce these risks.

**Level of Evidence:**

N/A.

**Abbreviations**
FIFA: Fédération Internationale de Football Association
IR: incidence rate
MH: match hours

## INTRODUCTION

In recent years, preventive medicine has gained increasing importance, particularly within sports medicine [8]. Recognising situations that pose a risk for injury is crucial for implementing effective preventive strategies. Addressing this requirement, the Fédération Internationale de Football Association (FIFA) introduced standardised definitions for ‘potential injury’ and ‘potential head injury’, thereby broadening the understanding of injury mechanisms to include at‐risk situations to further inform prevention strategies [2, 28]. These definitions enable more consistent assessments and also support meaningful comparisons across different sports disciplines.

Although beach soccer and futsal are variants of football and share some similarities [17], these sports differ substantially in several key aspects, including pitch dimensions, playing surfaces, movement patterns, playing style and exposure time calculation [9, 10, 16]. It is likely that these differences influence both the frequency and nature of head injuries observed during match‐play, however, to date, no known study has directly compared these sports, with data only being published in each sport separately [22, 28, 32]. For instance, a video analysis study of the 2022 FIFA World Cup, using data from FIFA match analysts, reported an average of 2.3 potential head injuries per match, corresponding to an incidence rate (IR) of 68.8 injuries per 1000 match hours (MH) [28]. Notably, 89% of these injuries resulted from opponent direct contact, 31% occurred during aerial duels, and 23.5% (IR of 16.2 injuries/1000 MH) were assessed by medical staff on the pitch [28]. A beach soccer study evaluating specifically potential head injuries that were evaluated by medical staff reported an average of 0.4 potential head injuries per match, yielding an IR of 68.3/1000 MH [22], nearly four times higher than the rate observed in football [28]. Among these, 74.8% involved opponent direct contact, 38% occurred during heading actions, and 23.8% associated with overhead kicks. In total, 15.3% demonstrated visible signs of possible concussion, and 9.9% led to permanent player substitutions. A recent study of the 2021 FIFA Futsal World Cup evaluating all head contact events found an average of 0.7 potential head injuries per match (IR of 108 injuries/1000 MH), of which 33% (IR of 35.6 injuries/1000 MH) were assessed by medical staff, and 60.5% were caused by upper extremity contact [32].

Repeated head impacts have been associated with a range of short‐ and long‐term health consequences, with the potential for players to be concussed from a direct or indirect force to the head [27]. Furthermore, it has been shown that recurrent concussions may be associated with more severe symptoms [38], prolonged recovery [6], persistent neurocognitive deficits [23] and an increased risk of a subsequent concussion or a secondary musculoskeletal injury [30]. From a clinical perspective, these consequences highlight not only the immediate need to recognise and manage concussion effectively, but also the broader imperative of reducing preventable head impacts that may lead to concussion [8]. Mapping the incidences and characteristics of potential head injuries in different football disciplines is therefore essential to raise awareness, guide rule enforcement and inform sport‐specific prevention strategies [8].

Potential head injuries encompass a range of diagnoses, such as contusions and lacerations, but concussion remains one of the most concerning [27]. In professional sports, sideline medical staff increasingly rely on video analysis to identify subtle indications of possible concussion that might go unnoticed during live play. Supporting this approach, six video signs of a possible concussion have been proposed by seven major sports organisations (e.g., the Australian Football League, World Rugby); however, experts from football were not included [7]. FIFA Medical has expanded on the previously recommended visible signs of possible concussion by introducing several new indicators and adapting them to football through a red and yellow flag classification system [11]. Red flags signal that the player is very likely to have a concussion requiring immediate substitution, while yellow flags indicate the need for clinical assessment (if not already performed) [11].

Although a few video analysis studies have examined potential head injuries in football, futsal and beach soccer [22, 28, 32], inconsistencies in definitions ‐ such as variations in the term ‘potential head injury’—and differences in exposure time have limited the comparability of findings across studies [1, 20, 33]. Moreover, the consistency and practical application of FIFA's flag criteria across football disciplines remain largely unexplored [11]. To address this knowledge gap, the present study systematically evaluates all potential head injury events across three international, professional men's tournaments: the 2022 FIFA World Cup, the 2024 FIFA Futsal World Cup and the 2024 FIFA Beach Soccer World Cup using a standardised video analysis protocol.

## METHODS

This cross‐sectional video analysis study included a total of 148 matches from across the 2022 FIFA World Cup (64 matches), the 2024 FIFA Futsal World Cup (52 matches) and the 2024 FIFA Beach Soccer World Cup (32 matches). Only men's tournaments were included in this video analysis study as no women's equivalent tournament currently exists for beach soccer and the first FIFA Women's Futsal World Cup is scheduled later in 2025. Match footage was obtained via the FIFA Football Data Platform (https://fdp.fifa.org). As the study was based on video analysis and did not involve the collection of personal or identifiable player data, an ethics exemption was granted by Swiss Ethics (BASEC‐Nr.: Req‐2023‐00635).

### Review procedures

Aligned with the medical extension of the FIFA Football Language framework, a potential head injury was defined as ‘any instance in which a player remained on the ground for more than 5 s and/or requested medical attention, following an impact involving the head’ [2, 28]. Impacts to the neck were also included. Accordingly, all beach soccer (Y.L. and O.K.) and futsal (Y.L. and L.K.) match footage was independently reviewed by two investigators to identify potential head injuries. Timestamps of potential head injuries at the 2022 FIFA World Cup were obtained from FIFA match analysts [28] and FIFA injury spotters, and reviewed by the two investigators (Y.L. and O.K.). Disagreements regarding whether an event qualified as a potential head injury were resolved through consultation with a third investigator (O.K. or L.K.), with final decisions being reached by consensus. Additionally, the adjusted exposure time was calculated for each match as the total playing time, defined as the duration from the initial to the final whistle. Only the durations of timeouts in futsal were subtracted from the exposure time analysis, as no competition takes place during these periods. All investigators were sports medicine specialists with a minimum of seven years of experience in sports injuries and all co‐authors had experience in evaluating injuries through video analysis.

### Coding procedures

All potential head injuries were independently evaluated using Kinovea (v0.9.5) with frame‐by‐frame analysis and coded based on predefined variables. Each potential head injury was evaluated using at least three different camera angles: program feed, tactical wide and high behind (right or left, depending on proximity to the potential event).

### Variables

The variables were grouped into three main categories: occurrence of potential head injury, player actions and outcomes [29]. Occurrence of potential head injury included contact mechanism, location of potential head injury and location of contact on the other player. Player actions included potentially injured player action, ball possession, player intent, contest nature, protective positioning and involvement of overhead kicks. Finally, outcomes included medical assessment, red and yellow flags of a possible concussion, the outcome of the potential head injury and referee decision.

Red and yellow flag classifications followed the criteria from FIFA's Injury Spotter Manual [11], which has been implemented in most FIFA football tournaments since 2022. Accordingly, the presence of one or more red flags indicate that the player has likely sustained a concussion and should be immediately substituted regardless of medical assessment findings, while the presence of one or more yellow flags indicate the need for clinical assessment (if one has not yet been conducted) [11]. Red flags included tonic posturing, motor incoordination, no protective action during fall (floppiness), impact seizure/convulsion and visible signs of potential concussion after return to play—after assessment. Yellow flags included blank/vacant look, lying motionless, disorientation, high force impact, dual head impact, face or scalp bleeding, visible signs of potential concussion after return to play without assessment [11]. Due to the absence of individual player tracking, the presence of ‘visible signs of potential concussion after return to play’ could not be evaluated using this video analysis methodology. A detailed list of all operational definitions used in this study is available in Supporting Information S1: Table S1.

### Data analysis

Statistical analyses were conducted using SPSS software (version 21.0; IBM Corp.). Categorical variables are presented as frequencies and percentages. For continuous variables, data are summarised using the mean and standard deviation or the median and interquartile range, depending on the distribution. Normality of the data distribution was assessed using skewness and kurtosis values (acceptable range between –2 and +2) and confirmed with the Shapiro–Wilk test. Chi‐square test was performed for comparisons of categorical variables, and effect sizes were calculated using Cramer's V [3]. The interrater reliability for all variables coded by two independent raters across all potential head injuries was evaluated using Cohen's Kappa. Agreement levels were categorised as follows: 0.81–1.00 indicated almost perfect agreement, 0.61–0.80 substantial, 0.41–0.60 moderate, 0.21–0.40 fair, 0.01–0.20 slight and values ≤ 0 indicated no agreement [24].

Official exposure time for each sport was calculated using the formula: (number of matches × match duration in minutes × number of players)/60. IRs were calculated per 1000 MH (using number of potential head injury situations/match exposure time × 1000) and 1000 player hours (number of potential head injury situations/match exposure time × 1000 × number of players) [36]. Similar calculations were performed according to adjusted exposure time.

## RESULTS

All variables demonstrated substantial to almost perfect agreement interrater reliability, with Cohen's kappa values of 0.71 or above (Supporting Information S1: Table S2).

### Potential head injuries

Of the 148 matches across all three sports, five matches included extra time in football, one in futsal and eight in beach soccer, thus the total official exposure time was 2167 MH for football, 347 MH for futsal and 196 MH for beach soccer. A total of 395 potential head injuries were identified: 186 in football, 86 in futsal and 123 in beach soccer. No potential head injuries occurred in four football matches (6.3%), 11 futsal matches (21.2%) and one beach soccer match (3.1%). The mean number of potential head injuries per match was 2.9 ± 1.9 (range: 0–8)—football, 1.6 ± 1.3 (range: 0–5)—futsal and 3.8 ± 2.1 (range: 0–9)—beach soccer, corresponding to IR of 85.8 (football), 247.8 (futsal) and 627.5 (beach soccer) potential head injuries/1000 MH.

### Adjusted exposure times and incidence rates

The mean adjusted exposure time, reflecting the total playing time per match was 103 min 45 s ± 9 min 45 s (range: 95 min 52 s–141 min 21 s) for football, 81 min 09 s ± 8 min 52 s (range: 56 min 25 s–107 min 35 s) for futsal and 60 min 12 s ± 5 min 49 s (range: 50 min 17 s–71 min 24 s) for beach soccer, corresponding to 2434, 703 and 321 MH, respectively. The adjusted IR were 76.4 (football), 122.3 (futsal) and 383.2 (beach soccer) potential head injuries/1000 MH.

### Distribution of potential head injuries

Four potential head injuries (two in football and two in futsal) were excluded from the analysis: two did not occur during the match, one had limited camera footage, and the other resulted from a foreign object thrown from the stands during goal celebration.

Goalkeepers accounted for 3.2% of potential head injuries in football, 19% in futsal, and 8.1% in beach soccer, corresponding to IR of 2.8, 46.1 and 51/1000 MH, respectively.

**Figure 1:**
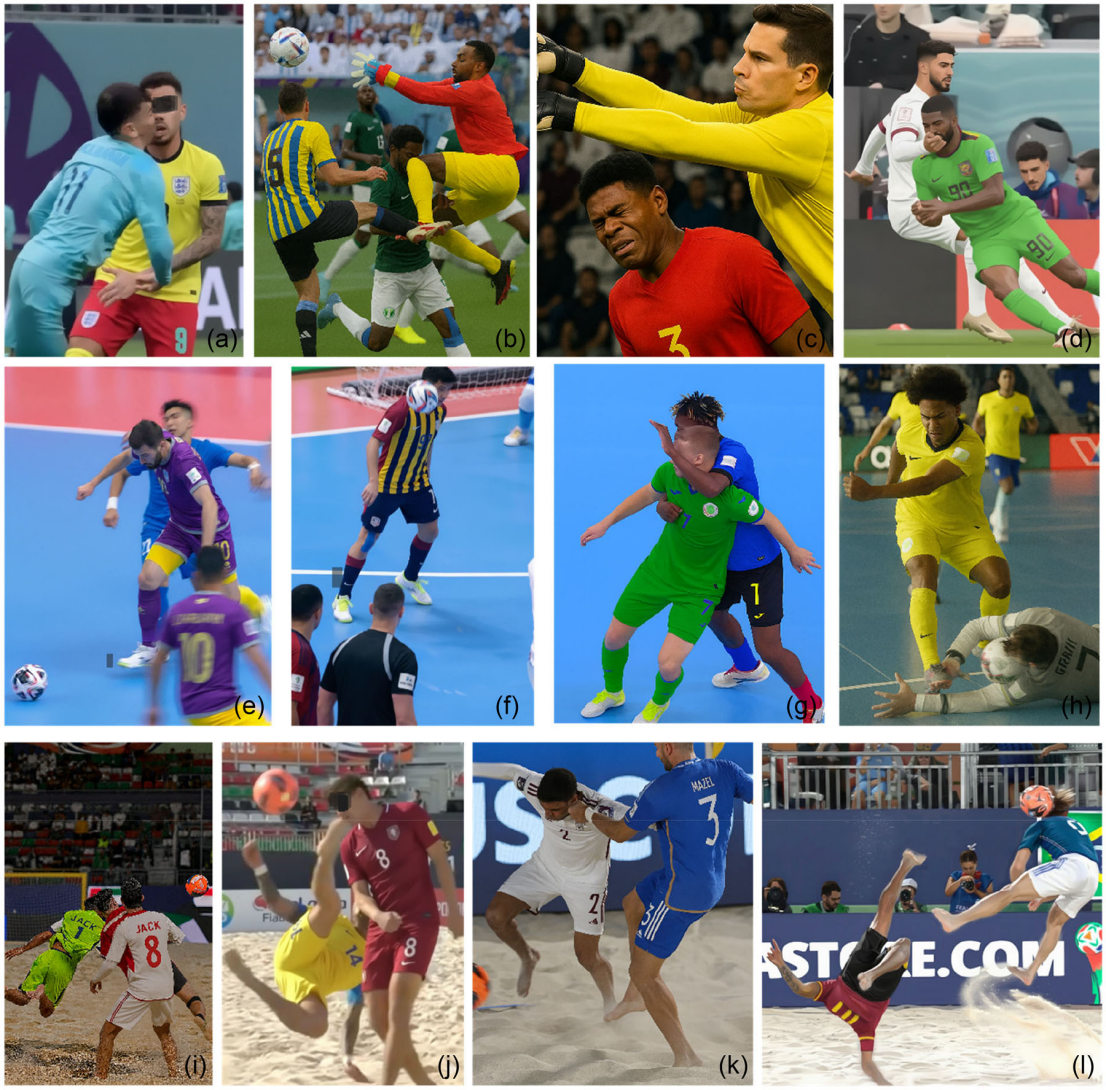
Occurrences and contact mechanism of potential head injuries in football, futsal and beach soccer. Football: (a) Opponent direct contact (head‐to‐head), (b) teammate contact (knee‐to‐head), (c) opponent direct contact (elbow‐to‐head), (d) opponent direct contact (forearm‐to‐head). Futsal: (e) Opponent direct contact (elbow‐to head), (f) unintentional ball contact (ball‐to head), (g) opponent direct contact (forearm‐to head), (h) goalkeeper unintentional ball contact (ball‐to head). Beach soccer: (i) Opponent direct contact (head‐to‐head), (j) opponent direct contact (foot‐to‐head), (k) opponent direct contact (hand‐to‐head), (l) unintentional ball contact (ball‐to‐head), overhead kick related. ChatGPT and Google Gemini were used to create images of common mechanisms based on match footage.

The distribution of potential head injuries according to mechanism, location and player actions were significantly different between disciplines (*p* = 0.04, Cramer's V = 0.14; *p* < 0.001, Cramer's *V* = 0.27; *p* < 0.001, Cramer's *V* = 0.32; respectively). The predominant mechanism across all disciplines was opponent direct contact, accounting for 81.5% of all incidents in football, 76.2% in futsal and 78.9% in beach soccer. While ball‐related contacts accounted for 7.1% of potential head injuries in football, this proportion was higher in futsal (19.1%) and in beach soccer (17.1%), corresponding to 6.0, 46.1 and 107.1 potential head injuries/1000 MH, respectively (Tables 1 and 2). Head‐to‐head contact was the most common mechanism of a potential head injury in football (21.2%), whereas it was not observed in futsal and constituted 7.3% of all potential injuries in beach soccer. The most common type of contact in both futsal and beach soccer was hand‐to‐head (33.3% and 19.1%, respectively) followed by ball‐to‐head (18.7% and 17.1%, respectively, with these percentages including both unintentional ball‐to‐head impacts and headers). Aerial duel was the most common player action in both football (45.1%) and beach soccer (22.8%), but not in futsal (7.1%). In futsal, the most common player action was blocking actions accounting for 22.6% of potential head injuries in futsal and 17.1% in beach soccer. While no potential head injuries related to overhead kicks were observed in football and futsal, 17.9% of such injuries in beach soccer were related to overhead kicks, corresponding to an IR of 122.2/1000 MH. The distribution of potential head injuries across different football disciplines is presented in Table 1 and Supporting Information S1: Table S3, and their IRs are shown in Table 2. Figure 1 presents the four most frequently observed occurrences and contact mechanisms for each discipline.

**Table 1:** The distribution of potential head injury variables according to different football disciplines.

Variables	Football	Futsal	Beach soccer
	% (*n*)	% (*n*)	% (*n*)
Occurrence of injury			
Contact mechanism			
Direct contact	97.8 (180)	98.8 (83)	99.2 (122)
Opponent contact	81.5 (150)	76.2 (64)	78.9 (97)
Unintentional ball contact	6 (11)	16.7 (14)	13.8 (17)
Teammate contact	5.4 (10)	2.4 (2)	‐
Other^a^	6.5 (12)	5.9 (5)	6.5 (8)
Unclear	0.5 (1)	‐	0.8 (1)
Difference between distributions	*p* = 0.04, Cramer's *V* = 0.14		
Location of contact on the other player			
Head‐to head	21.2 (39)	‐	7.3 (9)
Elbow‐to head	17.9 (33)	15.5 (13)	13 (16)
Hand‐to head	14.7 (27)	33.3 (28)	18.7 (23)
Forearm‐to head	10.9 (20)	10.7 (9)	13 (16)
Ball‐to head	7.1 (13)	19.1 (16)	17.1 (21)
Foot‐to head	2.7 (5)	‐	6.5 (8)
Other^a^	26.1 (48)	21.4 (18)	19.5 (24)
Unclear	2.2 (4)	‐	3.3 (4)
Difference between distributions	*p* < 0.001, Cramer's *V* = 0.27		
Player action			
Aerial duel	45.1 (83)	7.1 (6)	22.8 (28)
Ball progression	11.4 (21)	17.9 (15)	8.1 (10)
Other duels	9.2 (17)	19 (16)	19.5 (24)
Attempt at goal	6 (11)	2.4 (2)	4.9 (6)
Pressing	5.4 (10)	9.5 (8)	4.1 (5)
Block	3.8 (7)	22.6 (19)	17.1 (21)
Tackle	1.6 (3)	6 (5)	8.9 (11)
Other^a^	14.7 (27)	15.5 (13)	13 (16)
Unclear	2.7 (5)	‐	1.6 (2)
Difference between distributions	*p* < 0.001, Cramer's *V* = 0.32		
Overhead kick			
No	100 (184)	100) (84)	82.1 (101)
Yes	‐	‐	17.9 (22)
The outcome of the injury			
No medical evaluation	78.2 (144)	69 (58)	82 (101)
Evaluated and returned to match	20.7 (38)	29.7 (25)	14.7 (18)
Evaluated and substituted	1.1 (2)	‐	3.3 (4)
Unclear	‐	1.2 (1)	‐
Difference between distributions	*p* = 0.058, Cramer's *V* = 0.14		
Total	100 (184)	100 (84)	100 (123)

*Note*: Other in occurrence includes ground contact and header, in location of contact with the other player, it includes arm‐to‐head, elbow‐to‐neck, forearm‐to‐neck, hand‐to‐neck, object‐to‐head, hand‐to‐neck, hip‐to‐head, knee‐to‐head, leg‐to‐head, shoulder‐to‐head, thigh‐to‐head, ground‐to‐head and trunk‐to‐head; in player action, it includes pushing on, offering to receive, pass and other actions.

^a^ Variables with a frequency of less than 5% were grouped under ‘other’.

**Table 2:** The incidence rates of potential head injury variables according to different football disciplines.

Variables^a^	Football	Futsal	Beach soccer
	Incidence rate (1000 MH)	Incidence rate (1000 MH)	Incidence rate (1000 MH)
Occurrence of injury			
Contact mechanism			
Direct contact			
Opponent contact	69.2	184.4	494.8
Unintentional ball contact	5	40.3	86.7
Teammate contact	4.6	5.7	‐
Location of contact on the other player			
Head‐to head	17.9	‐	45.9
Elbow‐to head	15.2	37.4	81.6
Hand‐to head	12.4	80.6	117.3
Forearm‐to head	9.2	25.9	81.6
Ball‐to head	5.9	46.1	107.1
Foot‐to head	2.3	‐	40.8
Player action			
Aerial duel	38.3	17.2	142.8
Ball progression	9.6	43.2	51
Other duels	7.8	46.1	122.4
Attempt at goal	5	5.76	30.6
Pressing	4.6	23	25.5
Block	3.2	54.7	107.1
Tackle	1.3	14.4	56.1
Overhead kick			
No	84.9	242	515.3
Yes	‐	‐	112.2
Red flags			
No	83	242	627.5
Yes	1.8	‐	‐
Yellow flags			
No	71	219	500
Yes	13.8	23	127.5

Abbreviation: MH, match hours.

^a^ Excluded from analysis less than 5%, except for red flags.

Forty potential head injuries (21.8%) were medically assessed in football, 25 (31%) in futsal and 22 (18%) in beach soccer, with a mean assessment duration of 53 ± 70, 41 ± 37 and 35 ± 19 s, respectively. On average, players who were not substituted returned to the match 32 ± 33 s in football, 29 ± 21 in futsal and 32 ± 30 in beach soccer after a potential head injury incident was observed.

### Visible signs of possible concussion and medical assessment

Four red flags (2.2%) were identified in football, corresponding to an IR of 1.8 red flags/1000 MH and 0.08/1000 player hours. Of these red flags, three (75%) were motor incoordination and one (25%) was no protective action during fall (floppiness). All four potential head injuries with red flags were medically evaluated; with two (50%) players being immediately substituted, while the other two returned to the match following medical assessment. No red flags were observed in futsal or beach soccer.

Among potential head injuries, 30 (16.3%) in football, 8 (9.4%) in futsal and 25 (20.3%) in beach soccer involved yellow flags, corresponding to IR of 13.8, 23.0 and 127.5/1000 MH and 0.6, 2.3 and 12.7/1000 player hours, respectively. High force impact was the most common yellow flag in football (36.5%), futsal (33.3%) and beach soccer (43.3%). The distribution of red and yellow flags, and outcomes of these possible concussions are presented in Table 3.

**Table 3:** The distribution of red and yellow flags and subsequent outcomes following a head impact event.

Variables	Football	Futsal	Beach soccer
	% (*n*)	% (*n*)	% (*n*)
Potential head injuries with red flags	100 (4)	‐	‐
One red flag	100 (4)	‐	‐
Motor incoordination	75 (3)	‐	‐
Floppiness	25 (1)	‐	‐
The outcome of the potential head injury	100 (4)		
Evaluated and returned to match	50 (2)	‐	‐
Evaluated and substituted	50 (2)	‐	‐
No medical evaluation	‐	‐	‐
Potential head injuries with yellow flags	100 (30)	100 (8)	100 (25)
One yellow flag	73.3 (22)	87.5 (7)	80 (20)
High force impact	26.7 (8)	25 (2)	32 (8)
Lying motionless	16.7 (5)	12.5 (1)	16 (4)
Dual head impact	13.3 (4)	37.5 (3)	24 (6)
Face or scalp bleeding	13.3 (4)	12.5 (1)	8 (2)
Disorientation	3.3 (1)	‐	‐
Two yellow flags	20 (6)	12.5 (1)	20 (5)
High force impact and dual impact	10 (3)	‐	20 (5)
High force impact and lying motionless	6.7 (2)	‐	‐
Dual impact and disorientation	3.3 (1)	‐	‐
High force impact and blank/vacant look	‐	12.5 (1)	‐
Three or more yellow flags	6.7 (2)	‐	‐
High force impact, face or scalp bleeding and	3.3 (1)	‐	‐
disorientation			
High force impact, dual impact, lying motionless	3.3 (1)	‐	‐
and face or scalp bleeding			
All yellow flags	100 (41)	100 (9)	100 (30)
High force impact	36.6 (15)	33.3 (3)	43.3 (13)
Dual head impact	21.9 (9)	11.1 (1)	26.7 (8)
Lying motionless	19.5 (8)	33.3 (3)	23.3 (7)
Face or scalp bleeding	14.6 (6)	11.1 (1)	6.7 (2)
Disorientation	7.3 (3)	‐	‐
Blank/vacant look	‐	11.1 (1)	‐
The outcome of the potential head injury	100 (26)^a^	100 (8)	100 (25)
No medical evaluation	53.8 (14)	12.5 (1)	56 (14)
Evaluated and returned to match	46.2 (12)	75 (6)	36 (9)
Evaluated and substituted	‐	12.5 (1)	8 (2)

^a^ As four potential head injuries in football had both red and yellow flags, these four yellow flags were excluded from analysis.

## DISCUSSION

This study highlights key differences in potential head injury patterns across football, futsal and beach soccer. IRs were approximately three and seven times higher in futsal and beach soccer than in football, decreasing to 1.6 and five times higher, respectively, after exposure adjustment. Although direct opponent contact was the most frequently observed mechanism of a potential head injury in all three disciplines, in futsal and beach soccer unintentional ball‐related head impacts and overhead kicks resulted in more potential head injury events than in football. Additionally, while the proportion of visible signs of possible concussion was similar in football and beach soccer, the IR was nine times higher in beach soccer. Almost half of these events in football and beach soccer were not medically assessed.

The higher head injury rates in futsal and beach soccer compared to football have also been observed when comparing previous studies which have explored potential head injuries in each of the three disciplines in isolation [22, 28, 32]. However, differences in methodology limits comparability between studies for each of these disciplines [20, 22, 32]. By using standardised definitions and comprehensive video analysis across all matches, the present study provides the first direct comparison in professional men's tournaments, confirming that potential head injury rates in beach soccer and futsal were 7.3 and 2.8 times higher than in football. Moreover, the findings indicate that using official exposure time results in an overestimation of futsal injuries by 1.8 times and beach soccer injuries by 1.5 times compared with IR calculated using adjusted exposure time. Since game‐play and player actions continue during periods when the ball is out of play, injuries may still occur; therefore, these durations should be considered for inclusion in the exposure time. Doing so enables more accurate estimation of injury IR, particularly in futsal and beach soccer, and facilitates more appropriate cross‐disciplinary comparisons. These results can also be extrapolated to the development of prevention strategies and optimal assessment of performance‐related outcomes. Importantly, given the potentially short‐ and long‐term consequences of potential head injuries, including concussion, mapping their incidence and patterns across disciplines is essential to raise awareness, inform clinical practice and guide targeted strategies to decrease their occurrence.

Several contextual and structural differences between football, futsal and beach soccer likely contribute to the higher IR of potential head injuries in futsal and beach soccer. A key factor is the size of the playing area available per player, which is smaller in futsal and beach soccer at, approximately one‐third of that in the football [9, 10, 16], thereby increasing the frequency of close‐range interactions and physical contests. Previous studies have reported that this increased player density can lead to more frequent body contact and elevated injury risk [2, 19]. The present data appear to support this finding, showing that the incidence of other/physical duels was markedly higher in futsal and beach soccer, particularly involving upper extremity contact, which was 3.5 (futsal) and 7 (beach soccer) times more frequent when compared to football. To reduce the increased risk of potential head injuries from more frequent physical contact in futsal and beach soccer, limiting unnecessary duels and enforcing stricter rules may be beneficial [4], particularly regarding the use of upper extremities and high‐risk challenges.

One likely explanation for the much higher incidence of potential head injuries in beach soccer is the uneven sand surface, which can cause the ball to behave unpredictably when played on the ground. This likely prompts players to favour aerial play [18], increasing the risk of head impacts through player‐to‐player direct contact when players compete for an aerial ball. The present findings showed that IRs of potential head injuries resulting from aerial duels in beach soccer were nearly four times higher than in football and 8.5 times higher than in futsal. Moreover, it is possible that increased aerial play would lead to a higher number of headers. While header rates in professional men's football and futsal are reportedly comparable (IR: 2509 vs. 2375/1000 MH) [31, 32], such data are lacking for beach soccer, with these data not collected as part of this study. However, while the small number of potential head injuries related to headers limits accurate interpretation and renders the findings somewhat speculative, the IR in beach soccer was 22 times higher than in football (20.4 vs. 0.9/1000 h) and 3.5 times higher than in futsal (20.4 vs. 5.8/1000 h), warranting further investigation given the potential association between headers short‐ and long‐term neurological consequences [15, 26, 37].

The uneven and unpredictable nature of the sand surface also likely increases the frequency of overhead kicks, another form of aerial play that has been previously associated with potential head injuries in beach soccer [20]. In the present study, nearly 20% of potential head injuries in beach soccer were associated with overhead kicks, a rate higher than the overall incidence of potential head injuries in football [28]. Furthermore, players exhibited protective actions in 44.4% of potential head injuries related to overhead kicks, compared to only 14.7% in those not related to overhead kicks, indicating that protective positioning in overhead kick‐related situations remains insufficient. Additionally, in nearly half of the potential head injuries related to overhead kicks, defenders turned their backs towards the attacker, likely as a protective mechanism intended to shield their head and face from contact, yet this often prevented them from tracking both the ball and the other player's actions leading to contact to the side or back of their head instead. Given that other potential head injuries resulted from opponent direct contact, the need for stricter enforcement of the rule that prohibits contact with players performing overhead kicks is recommended to reduce player‐to‐player contact.

Another potential contributing factor is the increased offensive intensity in futsal and beach soccer. In the final matches of the respective tournaments, goal attempts occurred at rates of 2.3 and 2.8 per minute in futsal and beach soccer [13, 14], almost 10 times the rate in football [12]. This high‐intensity offensive tempo increases players' exposure to ball‐related impacts, particularly for goalkeepers and defenders engaged in blocking actions. Correspondingly, potential head injuries involving goalkeepers were 16 and 18 times more common in futsal and beach soccer, respectively. Additionally, the design of the futsal ball, which is smaller but of similar weight to a standard football [10, 16], may produce higher impact forces in a more compact space during ball‐related impacts, particularly unintentional ball‐to‐head impacts. While this biomechanical aspect has yet to be systematically studied, it may present as a plausible mechanism for increased head injury risk in futsal from ball‐related impacts. Training programs that include specific drills on safe blocking and tackle techniques, particularly for goalkeepers, may help reduce the risk of head injuries during high‐intensity goal attempts and other ball‐related activities.

Although the proportion of visible signs of possible concussion was lower in futsal and slightly higher in beach soccer when compared to football, the overall IRs were 1.5 (futsal) and 9 (beach soccer) times higher. However, while red flags were observed in 2.2% of football potential head injury incidents, no red flags were observed in futsal and beach soccer, suggesting that concussion risk may be higher in football (although the number of incidents are very small making this suggestion highly speculative). It has been reported that high‐force head impacts are more likely to occur when two or more players collide during high‐speed running on larger pitches [5, 25]. Risk of injury may also be increased when player contact is not anticipated. Interestingly, in football there were two potential head injuries involving a contested situation resulting in a red flag of a possible concussion where the potentially injured player in both incidents appeared unaware of the approaching player, emphasising the role of observation, scanning, and body positioning to promote spatial and player awareness. Additionally, more than half of the cases showing visible signs of possible concussion in football and beach soccer were not medically assessed, and two (50%) of possible concussion with red flags returned to play following medical assessment, highlighting the need to continue to promote concussion recognition and management [27]. Video review may allow for the systematic detection of visible signs of possible concussion, such as motor incoordination or lack of protective action during a fall, that may be missed in real time, thereby supporting sideline medical decision‐making. However, video analysis alone cannot be used to determine injury diagnoses, and should only be used as an adjunct to clinical assessment to support on‐field or postmatch concussion recognition and facilitate timely player management across football disciplines.

From a clinical perspective, the present findings provide important insights for practitioners. Identifying that most potential head injuries arise from direct contact in contested situations, and that overhead kicks represent a unique mechanism in beach soccer, may assist clinicians in recognising match situations with an elevated risk of head injuries, and assist the development of strategies to prepare players to protect themselves. While the majority of visible signs in this study were categorised as yellow flags, meaning that these players were less likely to be diagnosed with a concussion when compared with players exhibiting visible signs categorised red flags, the observation of a yellow flag following a head impact should lead to a medical assessment. The finding that a considerable proportion of players showing visible signs categorised as yellow flags were not medically assessed highlights the ongoing need for vigilance among medical staff and the application of standardised video criteria to support rapid on‐field clinical decision‐making. For coaches, understanding the influence of playing style and specific actions such as blocking or aerial duels can inform training modifications and tactical adjustments to reduce head injury risk. Together, these insights can contribute to refining prevention strategies tailored to the specific demands of football, futsal and beach soccer.

Several limitations should be noted for this study. While an earlier study from the 2022 FIFA World Cup that used data collected by FIFA data analysts reported 149 potential head injuries, a total of 186 incidents were reported using data from both FIFA analysts and FIFA injury spotters. Additionally, the potential head injury definition in this present study was broadened to include the neck. This has led to a higher number of identified incidents, underscoring the need for high inter‐rater reliability during data collection (inter‐rater reliability scored much higher in this study when compared with the analyst data) which may also be related to professional backgrounds (i.e., coders with medical backgrounds may be more adept at identifying potential injury situations than nonmedical coders). While video analysis is a reliable tool for identifying injury mechanisms [21, 34, 35], it cannot confirm clinical diagnoses. Thus, findings should be interpreted as possible, not verified, concussions [7]. The FIFA red/yellow flag classification is relatively new and lacks full validation. Future studies should integrate clinical evaluations and diagnoses. Inclusion of all matches in the respective tournaments enhances generalisability to professional men in each of these sports, but studies in women, youth and amateur levels are needed. Unlimited substitutions in futsal and beach soccer also made it challenging to evaluate injury outcomes, particularly towards the end of the match. These limitations should be considered when interpreting the findings and generalising to broader contexts.

## CONCLUSION

The present study demonstrates the importance of using adjusted exposure time when comparing potential head injury incidence across football, futsal and beach soccer. The findings demonstrate both shared mechanisms, such a direct opponent contact being most frequent, and discipline‐specific mechanisms, with unintentional ball‐to‐head impacts contributing substantially to the IR of potential head injuries in futsal and beach soccer, and overhead kicks posing a unique risk in beach soccer. A considerable proportion of incidents with visible signs of possible concussion (classified as yellow flags) were not medically assessed, underlining the potential of promoting visible signs following a head impact event to prompt on‐field medical assessment.

## AUTHOR CONTRIBUTIONS

**Yavuz Lima**: Conception and design, data acquisition, writing, proofreading. **Levend Karaçoban**: Internal review, data analysis, proofreading. **Andreas Serner**: Conception, internal review, data acquisition, proofreading. **Kerry Peek**: Internal review, data acquisition, proofreading, supervision. **Ogün Köyağasıoğlu**: Internal review, data acquisition, proofreading.

## CONFLICT OF INTEREST STATEMENT

The authors declare no conflicts of interest.

## ETHICS STATEMENT

As the study was based on video analysis and did not involve the collection of personal or identifiable player data, an ethics exemption was granted by Swiss Ethics (BASEC‐Nr.: Req‐2023‐00635).

## Supporting information

Supporting information.
