# Supplementary material for: Potential head injuries in men's football, futsal and beach soccer: Distinct exposure‐adjusted frequency and patterns from a comparative video analysis
Source: Knee Surg Sports Traumatol Arthrosc. 2025 Oct 27;34(3):1107–17. doi: 10.1002/ksa.70158 (PMC12948335; doi:10.1002/ksa.70158)
Supplement: Supplementary file 1 — Supporting information. [file KSA-34-1107-s001.docx]

**Supplementary Table 1. The reporting categories and definitions of potential head injuries used in the present study**

| **Category** | | **Definition** |
| --- | --- | --- |
|  | **Variable** |  |
| **Occurrence of potential head injury** | |  |
|  | **Contact mechanisms** | The mechanism of the potential head injury. |
|  | **Direct contact** |  |
|  | Opponent contact | Direct contact of the opponent to head. |
|  | Unintentional ball contact | Any instance of unintentional contact between a player's head and the ball. |
|  | Header | A deliberate action in which a player uses their head to intentionally alter the direction of the ball. |
|  | Teammate contact | Direct contact of the teammate to head. |
|  | Ground contact | Direct contact of the head to the ground. |
|  | **Indirect contact** | Injured player had a contact to a body part other than the head region. |
|  |  |  |
|  | **Location of contact** | The area of the head or any other part of the body where the player sustained the impact. Reported as following: Frontal, nasal, zygomatic, maxillary, mandibular, temporal, parietal, occipital, neck, shoulder, upper back, other. |
|  | **Location of contact on the other player** | The body part of the opposing player that resulted in the injury. Reported as following: Head, shoulder, upper arm, elbow, forearm, wrist/hand, trunk, hip, thigh, knee, leg, ankle/foot |
| **Player actions** | |  |
|  | **Potentially injured player action** | The injured player action |
|  | Aerial duel | A physical contest between at least two players for possession of the ball in the air, involving at least one player is off the ground. |
|  | Other duels | A physical contest between at least two players for winning or retaining the possession of the loose ball. |
|  | Block | Defensive action that a player uses their body to prevent the ball from reaching a target, often by positioning themselves in front of a shot, pass, cross, or clearance. |
|  | Attempt at goal | Player actions with the intention of scoring a goal |
|  | Ball progression | Moving the ball mostly towards the opposition’s goal area. |
|  | Tackle | Defensive action where a player attempts to win the ball by using body parts. |
|  | Offering to receive | Player action to make themselves available to receive a pass from a teammate, often by positioning their body clearly or calling for the ball. |
|  | Pressing | Defensive action to apply immediate pressure on an opponent by closing the space in between, aiming to force opponent to make mistakes. |
|  | Clearance | Player attempt to send the ball away from the current area, typically to relieve pressure or danger from the opposing team. |
|  | Pass | Sending the ball to a teammate using feet, head, or other body parts (excluding hands and arms) on the ground or aerially. |
|  | Pushing on | Offensive action that one or more players advance forward aggressively to get closer to opponent players. |
|  | **Overhead kick** | The situation in which any player performs or intent to perform an overhead kick at the time of potential head injury. |
|  | **Ball possession nature** | Control status of the ball during the potential injury event |
|  | Free ball | The ball is not under control of any player |
|  | Potentially injured player | The ball is under control of the potentially injured player |
|  | Uninjured player | The ball is under control of an uninjured player |
|  | Without ball | The player is potentially injured in a situation unrelated to the ball |
|  | **Contested nature** | Whether two or more players are competing for the ball |
|  | **Protective body positioning** | Whether the potentially injured player had adopted a protective body position prior to the potential injury event |
|  | No protective action | The player does not have any protective body positioning |
|  | Protective action - turns away | The player turns their body away from the ball or opponent for protection |
|  | Protective action – head | The player moves their head for protection |
|  | Protective action – arms | The player tries to cover their head with their arms for protection |
|  | **Interference** | Making an intervention during a contested situation with the intention of gaining possession of or playing the ball |
|  | Interference – pushed off the ball | Illegal use of force, typically with the hands or body, to displace an opponent from the ball. |
|  | No interference | The situation with no interference |
|  | Interference – held off the ball | Legal use of the body or arm to shield, gain possession of the ball, or maintain position without applying excessive force. |
|  | Interference – opposition other | Interference – other than push-off and held-off situations |
|  | Interference- teammate | The situation of teammate interference |
|  | **Player intent** | The player intent during a contested event. Reported as following: |
|  |  | Compete to win the ball – head |
|  |  | Compete to win the ball – feet |
|  |  | Compete to win the ball - other |
|  |  | Compete to win the position |
|  |  | Goalkeeper competing for aerial ball |
|  |  | Goalkeeper to win the ball – other |
|  |  | Interfere with opponent's action |
|  |  | No competition |
|  |  | The player already had the ball |
|  | **Potentially injured player awareness** | The potentially injured player's awareness of the other player during a contested event |
|  | **Obstruction** | Making an intervention during a contested situation without the intention of gaining possession of or playing the ball. Reported as following: no obstruction, obstructed by opponent, obstructed by teammate, and obstructed by multiple players. |
| **Outcomes** | |  |
|  | **Whether the match was stopped** | Whether the match was stopped or not due to a potential injury |
|  | **Whether the player was evaluated** | Whether the player was evaluated or not |
|  | **Who evaluated** | The evaluator. Reported as following: referee, medical staff, teammate, opponent player |
|  | **The outcome of the potential head injury** | The outcome of the potential head injury. Reported as following: no medical evaluation, evaluated medical staff and resume to the match, evaluated by medical staff and immediately substituted |
|  | **The duration of the medical assessment** | The duration between when the medical staff arrived and when the assessment of the potential head injury ended. |
|  | **Video signs of possible concussion** | Categorization of video observable red and yellow flags of a possible concussion following the potential injury event. |
|  | **Red flags** |  |
|  | Motor incoordination | The player has unstable or unsteady movements following the injury such as struggling to keep balance or get up, staggering, stumbling, or struggling. |
|  | Impact seizure | The player has involuntary asymmetric and irregular clonic contractions in axial or limb muscles following the injury. |
|  | Tonic posturing | The player has involuntary contractions of one or more limbs, that held the related body part in a stiff condition following the injury. |
|  | No protective action (floppiness) | The player falls to the ground without any protective movement following the injury such as extending limbs to minimise the ground reaction forces. |
|  | Vomiting | Any immediate or delayed vomiting following head impact. |
|  | **Yellow flags** |  |
|  | Lying motionless | The player lies without a significant purposeful movement, or does not react, respond or reply at least two seconds following the injury. |
|  | Blank and vacant look | The player exhibits emotionless abnormal facial expression environment following the injury. |
|  | Disorientation | The player appears disoriented in both movements and facial expressions. |
|  | Dual head impacts | Whether injured player had an additional head impact that occurred right after the initial potential head injury. Reported as yes or no. |
|  | High-force head impact | Events like a knee-to-head impact, where the head moves rapidly and uncontrollably. |
|  | Face or scalp bleeding | Whether the injured player had bleeding following the injury. Reported as yes or no. |
|  | **The referee decision** | The referee’s decision following the potential head injury event. Reported as following: no sanction, foul, card (red or yellow), and against foul |
| **Other** | |  |
|  | **The player** | Goalkeeper or outfielder player. |
|  | **The duration of the potential head injury event** | The time between a player had a potential head injury and resume to play (in cases where the player resumed playing) or the moment the player left the field (in cases where the player was unable to resume playing for a while or unable to return to the match) |
|  | **Team status** | Whether the team position of the potentially injured player. Reported as following: attack, defence, or in contest |

| **Supplementary Table 2. Interrater reliability of the variables** | | | | | | | | | | | | | |
| --- | --- | --- | --- | --- | --- | --- | --- | --- | --- | --- | --- | --- | --- |
| **Variable** | **Football** | | | | **Futsal** | | | | **Beach Soccer** | | | | |
|  | **Rater A** (n) | **Rater B** (n) | **Overall  Agreement** | **Kappa** (95% CI) | **Rater A** (n) | **Rater B** (n) | **Overall  Agreement** | **Kappa** (95% CI) | **Rater A** (n) | **Rater B** (n) | **Overall  Agreement** | **Kappa** (95% CI) |  |
| **Contact mechanism** |  |  | 94% | 0.83 [0.74-0.93] |  |  | 91.8% | 0.81 [0.68-0.94] |  |  | 91.1% | 0.78 [0.65-0.9] |  |
| Opponent contact | 146 | 147 |  |  | 62 | 60 |  |  | 94 | 93 |  |  |  |
| Unintentional ball contact | 11 | 10 |  |  | 14 | 14 |  |  | 17 | 17 |  |  |  |
| Header | 2 | 2 |  |  | 3 | 2 |  |  | 6 | 4 |  |  |  |
| Teammate contact | 13 | 10 |  |  | 3 | 6 |  |  | 1 | 4 |  |  |  |
| Ground contact | 7 | 7 |  |  | 1 | 1 |  |  | 4 | 4 |  |  |  |
| Other | - | - |  |  | 1 | 1 |  |  | - | - |  |  |  |
| Indirect contact | 3 | 3 |  |  | - | - |  |  | - | - |  |  |  |
| Unclear | 2 | 5 |  |  | - | - |  |  | 1 | 1 |  |  |  |
| **Location of contact on the other player** |  |  | 91.5% | 0.91 [0.87-0.96] |  |  | 80.5% | 0.79 [0.69-0.88] |  |  | 87.8% | 0.86 [0.8-0.93] |  |
| Head-to head | 36 | 37 |  |  | - | - |  |  | 10 | 10 |  |  |  |
| Elbow- to head | 31 | 35 |  |  | 12 | 9 |  |  | 15 | 16 |  |  |  |
| Wrist/hand- to head | 29 | 26 |  |  | 29 | 31 |  |  | 21 | 24 |  |  |  |
| Wrist/hand- to neck | - | - |  |  | - | - |  |  | 1 | 2 |  |  |  |
| Forearm- to head | 20 | 21 |  |  | 9 | 12 |  |  | 20 | 16 |  |  |  |
| Shoulder- to head | 11 | 8 |  |  | 4 | 3 |  |  | 3 | 2 |  |  |  |
| Foot- to head | 5 | 4 |  |  | - | - |  |  | 7 | 7 |  |  |  |
| Knee- to head | 4 | 6 |  |  | 2 | 2 |  |  | 3 | 2 |  |  |  |
| Trunk- to head | 5 | 4 |  |  | 2 | 2 |  |  | 3 | 4 |  |  |  |
| Thigh- to head | - | - |  |  | 3 | 2 |  |  | 4 | 4 |  |  |  |
| Leg- to head | 4 | 2 |  |  | 2 | 3 |  |  | 2 | 3 |  |  |  |
| Arm- to head | 5 | 7 |  |  | 1 | 1 |  |  | 3 | 2 |  |  |  |
| Hip- to head | - | - |  |  | - | - |  |  | 2 | 2 |  |  |  |
| Other | 10 | 10 |  |  | 4 | 4 |  |  | - | - |  |  |  |
| Unclear | 4 | 4 |  |  | - | - |  |  | 4 | 4 |  |  |  |
| **Location of contact** |  |  | 81.5% | 0.79 [0.73-0.86] |  |  | 86.9% | 0.85 [0.76-0.93] |  |  | 86.9% | 0.85 [0.79-0.92] |  |
| Temporal | 28 | 23 |  |  | 5 | 8 |  |  | 21 | 18 |  |  |  |
| Occipital | 22 | 20 |  |  | 9 | 7 |  |  | 19 | 21 |  |  |  |
| Frontal | 15 | 18 |  |  | 3 | 4 |  |  | 14 | 12 |  |  |  |
| Mandibular | 33 | 33 |  |  | 15 | 18 |  |  | 14 | 15 |  |  |  |
| Nasal | 10 | 10 |  |  | 11 | 14 |  |  | 8 | 8 |  |  |  |
| Parietal | 14 | 16 |  |  | 5 | 4 |  |  | 9 | 12 |  |  |  |
| Zygomatic | 27 | 24 |  |  | 26 | 20 |  |  | 18 | 15 |  |  |  |
| Maxillary | 10 | 17 |  |  | 7 | 6 |  |  | 5 | 8 |  |  |  |
| Neck | 7 | 6 |  |  | - | - |  |  | 3 | 2 |  |  |  |
| Upper back | 1 | 1 |  |  | - | - |  |  | - | - |  |  |  |
| Trunk | 2 | 2 |  |  | - | - |  |  | - | - |  |  |  |
| Unclear | 15 | 14 |  |  | - | - |  |  | 12 | 12 |  |  |  |
| **Player action** |  |  | 82.1% | 0.78 [0.71-0.84] |  |  | 64.3% | 0.77 [0.67-0.96] |  |  | 78.9% | 0.76 [0.67-0.84] |  |
| Aerial duel | 81 | 70 |  |  | 9 | 5 |  |  | 31 | 23 |  |  |  |
| Ball progression | 14 | 20 |  |  | 15 | 11 |  |  | 10 | 11 |  |  |  |
| Block | 9 | 9 |  |  | 20 | 18 |  |  | 24 | 22 |  |  |  |
| Tackle | 7 | 3 |  |  | 4 | 5 |  |  | 9 | 11 |  |  |  |
| Clearance | 10 | 8 |  |  | - | - |  |  | 6 | 6 |  |  |  |
| Pass | 10 | 7 |  |  | 3 | 3 |  |  | 5 | 5 |  |  |  |
| Pressing | 8 | 9 |  |  | 10 | 10 |  |  | 5 | 4 |  |  |  |
| Offering to receive | 9 | 8 |  |  | 4 | 4 |  |  | 3 | 5 |  |  |  |
| Other duel | 16 | 21 |  |  | 11 | 22 |  |  | 19 | 25 |  |  |  |
| Pushing on | 2 | 2 |  |  | - | - |  |  | 2 | 3 |  |  |  |
| Attempt at goal | 10 | 15 |  |  | 2 | 2 |  |  | 7 | 6 |  |  |  |
| Other | 1 | 1 |  |  | 6 | 4 |  |  | - | - |  |  |  |
| Unclear | 7 | 11 |  |  | - | - |  |  | 2 | 2 |  |  |  |
| **Overhead kick** |  |  | - | - |  |  | - | - |  |  | 97.6% | 0.92 [0.83-0.98] |  |
| Yes | - | - |  |  | - | - |  |  | 23 | 22 |  |  |  |
| No | 184 | 184 |  |  | 984 | 84 |  |  | 100 | 101 |  |  |  |
| **Contested nature** |  |  | 98.4% | 0.83 [0.65-0.98] |  |  | 95.2% | 0.83 [0.67-0.99] |  |  | 96.7% | 0.82 [0.64-0.99] |  |
| Contested | 175 | 174 |  |  | 68 | 72 |  |  | 111 | 111 |  |  |  |
| Uncontested | 9 | 10 |  |  | 16 | 12 |  |  | 12 | 12 |  |  |  |
| **Player awareness** |  |  | 91.3% | 0.79 [0.69-0.89] |  |  | 73.8% | 0.77[0.62-0.91] |  |  | 90.3% | 0.71 [0.55-0.87] |  |
| Yes | 146 | 135 |  |  | 67 | 59 |  |  | 96 | 107 |  |  |  |
| No | 25 | 36 |  |  | 3 | 11 |  |  | 17 | 6 |  |  |  |
| Unclear | 2 | 2 |  |  | - | - |  |  | - | - |  |  |  |
| **Ball possession** |  |  | 92.9% | 0.88 [0.81-0.94] |  |  | 71.4% | 0.77 [0.67-0.87] |  |  | 85.4% | 0.79 [0.71-0.88] |  |
| Free ball | 113 | 112 |  |  | 19 | 19 |  |  | 53 | 44 |  |  |  |
| Uninjured player | 22 | 18 |  |  | 34 | 29 |  |  | 35 | 37 |  |  |  |
| Injured player | 27 | 34 |  |  | 18 | 16 |  |  | 20 | 25 |  |  |  |
| Without ball | 22 | 20 |  |  | 13 | 20 |  |  | 15 | 17 |  |  |  |
| **Injured player intent during contested event** |  |  | 92.5% | 0.77 [0.69-0.84] |  |  | 87.1% | 0.87 [0.79-0.95] |  |  |  | 0.88 [0.92-0.95] |  |
| Compete to win the ball – head | 76 | 81 |  |  | 7 | 7 |  |  | 25 | 27 |  |  |  |
| Compete to win the ball – feet | 24 | 21 |  |  | 22 | 21 |  |  | 27 | 30 |  |  |  |
| The player has already had the ball | 24 | 23 |  |  | 16 | 16 |  |  | 21 | 17 |  |  |  |
| No competition | 36 | 32 |  |  | 16 | 16 |  |  | 25 | 25 |  |  |  |
| Goalkeeper competing for ball | 4 | 5 |  |  | 6 | 7 |  |  | 10 | 9 |  |  |  |
| Compete to win the ball – other | 9 | 11 |  |  | 2 | 2 |  |  | 4 | 4 |  |  |  |
| Interfere with opponent's action | - | - |  |  | 1 | 1 |  |  | 1 | 1 |  |  |  |
| Unclear | - | - |  |  | - | - |  |  | - | - |  |  |  |
| **Protective body positioning** |  |  | 94.2% | 0.83 [0.72-0.93] |  |  | 94.3% | 0.86 [0.72-0.99] |  |  | 82.9% | 0.89 [0.81-0.98] |  |
| No protective action | 154 | 150 |  |  | 66 | 68 |  |  | 89 | 89 |  |  |  |
| Protective action – arms | 9 | 6 |  |  | 4 | 2 |  |  | 4 | 4 |  |  |  |
| Protective action - turns away | 5 | 8 |  |  | - | - |  |  | 12 | 10 |  |  |  |
| Protective action - head | 3 | 4 |  |  | - | - |  |  | 6 | 8 |  |  |  |
| Unclear | 2 | 5 |  |  |  |  |  |  | 2 | 2 |  |  |  |
| **Obstruction** |  |  | 93.1% | 0.93 [0.89-0.97] |  |  | 90% | 0.86 [0.76-0.96] |  |  | 85.4% | 0.83 [0.72-0.95] |  |
| No obstruction | 134 | 134 |  |  | 44 | 51 |  |  | 93 | 95 |  |  |  |
| Yes - obstructed by opponent | 37 | 37 |  |  | 25 | 18 |  |  | 19 | 17 |  |  |  |
| Yes - obstructed by multiple players | - |  |  |  | 1 | 1 |  |  | - | - |  |  |  |
| Yes - obstructed by teammate | 2 | 2 |  |  | - | - |  |  | 1 | 1 |  |  |  |
| **Interference** |  |  | 93.6% | 0.95 [0.91-0.98] |  |  | 80% | 0.78 [0.67-0.88] |  |  |  | 0.78 [0.7-0.86] |  |
| Interference – pushed off ball | 91 | 95 |  |  | 30 | 25 |  |  | 55 | 50 |  |  |  |
| Interference – held off ball | 24 | 20 |  |  | 7 | 9 |  |  | 12 | 13 |  |  |  |
| No interference | 43 | 43 |  |  | 23 | 30 |  |  | 34 | 38 |  |  |  |
| Interference – opposition other | 5 | 4 |  |  | 8 | 4 |  |  | 12 | 12 |  |  |  |
| Interference- teammate | 9 | 10 |  |  | 2 | 2 |  |  | - | - |  |  |  |
| Unclear | 1 | 1 |  |  | - | - |  |  | - | - |  |  |  |
| **Outcomes** |  |  |  |  |  |  |  |  |  |  |  |  |  |
| **Red flags** |  |  | 98.4% | 0.72 [0.42-0.98] |  |  | - | - |  |  | - | - |  |
| Yes | 5 | 6 |  |  | - | - |  |  | - | - |  |  |  |
| No | 177 | 174 |  |  | 84 | 84 |  |  | 123 | 123 |  |  |  |
| **Yellow flags** |  |  | 95.1% | 0.95 [0.91-0.98] |  |  | 96.4% | 0.82 [0.63-0.99] |  |  | 93.5% | 0.81 [0.68-0.93] |  |
| Yes | 33 | 30 |  |  | 11 | 8 |  |  | 27 | 25 |  |  |  |
| No | 151 | 154 |  |  | 73 | 76 |  |  | 96 | 98 |  |  |  |
| **The outcome of the injury** |  |  | 96.2% | 0.88 [0.8-0.97] |  |  | 92.9% | 0.86 [0.75-0.98] |  |  | 95.1% | 0.85 [0.72-0.97] |  |
| Evaluated and continued the match | 37 | 34 |  |  | 22 | 27 |  |  | 19 | 19 |  |  |  |
| No medical evaluation | 145 | 148 |  |  | 61 | 56 |  |  | 100 | 100 |  |  |  |
| Evaluated and immediately substituted | 2 | 2 |  |  | 1 | 1 |  |  | 4 | 4 |  |  |  |
| **The referee decision** |  |  | 92.4% | 0.94 [0.91-0.97] |  |  | 88.1% | 0.81 [0.7-0.92] |  |  | 92.7% | 0.88 [0.8-0.96] |  |
| No sanction | 98 | 96 |  |  | 47 | 40 |  |  | 63 | 65 |  |  |  |
| Foul | 55 | 50 |  |  | 20 | 29 |  |  | 40 | 37 |  |  |  |
| Against foul | 9 | 18 |  |  | 2 | 2 |  |  | 6 | 6 |  |  |  |
| Card | 22 | 20 |  |  | 15 | 13 |  |  | 14 | 15 |  |  |  |
| **Team status** |  |  | 89.7% | 0.84 [0.77-0.9] |  |  | 92.3% | 0.91 [0.84-0.98] |  |  | 95.9% | 0.92 [0.86-0.99] |  |
| Defence | 71 | 74 |  |  | 47 | 42 |  |  | 72 | 68 |  |  |  |
| Attack | 79 | 78 |  |  | 27 | 33 |  |  | 46 | 49 |  |  |  |
| In contest | 34 | 32 |  |  | 10 | 9 |  |  | 5 | 6 |  |  |  |

**Supplementary Table 3. The distribution of additional potential head injury variables according to different football disciplines**

| **Variables** | | **Football** | **Futsal** | **Beach soccer** |
| --- | --- | --- | --- | --- |
|  | | ***% (n)*** | ***% (n)*** | ***% (n)*** |
| **Occurrence of injury** | |  |  |  |
|  | **Contact mechanism** |  |  |  |
|  | **Direct contact** | 97.8 (180) | 98.8 (83) | 99.2 (122) |
|  | Opponent contact | 81.5 (150) | 76.2 (64) | 78.9 (97) |
|  | Unintentional ball contact | 6 (11) | 16.7 (14) | 13.8 (17) |
|  | Teammate contact | 5.4 (10) | 2.4 (2) | - |
|  | Ground contact | 3.8 (7) | 1.2 (1) | 3.3 (4) |
|  | Header | 1.1 (2) | 2.4 (2) | 3.3 (4) |
|  | **Indirect contact** | 1.6 (3) | - | - |
|  | Other contact | - | 1 (1.2) | - |
|  | Unclear | 0.5 (1) | - | 0.8 (1) |
|  | Total | 100 (184) | 100 (84) | 100 (123) |
| **Location of contact** | |  |  |  |
|  | Mandibular | 19.6 (36) | 17.9 (15) | 11.4 (14) |
|  | Temporal | 14.7 (27) | 6 (5) | 16.3 (20) |
|  | Zygomatic | 13.6 (25) | 28.6 (24) | 13 (16) |
|  | Occipital | 12 (22) | 10.7 (9) | 15.4 (19) |
|  | Frontal | 9.2 (17) | 4.8 (4) | 9.8 (12) |
|  | Parietal | 6.5 (12) | 6 (5) | 8.9 (11) |
|  | Maxillary | 6.5 (12) | 8.3 (7) | 4.9 (6) |
|  | Nasal | 6 (11) | 14.3 (12) | 7.3 (9) |
|  | Neck | 3.8 (7) | 3.6 (3) | 2.4 (3) |
|  | Trunk | 1.1 (2) | - | - |
|  | Upper back | 0.5 (1) | - | - |
|  | Unclear | 6.5 (12) | - | 13 (10.6) () |
|  | Total | 100 (184) | 100 (84) | 100 (123) |
| **Location of contact on the other player** | |  |  |  |
|  | Head-to head | 21.2 (39) | - | 7.3 (9) |
|  | Elbow-to head | 17.9 (33) | 15.5 (13) | 13 (16) |
|  | Hand-to head | 14.7 (27) | 33.3 (28) | 18.7 (23) |
|  | Forearm-to head | 10.9 (20) | 10.7 (9) | 13 (16) |
|  | Ball-to head | 7.1 (13) | 19.1 (16) | 17.1 (21) |
|  | Shoulder-to head | 4.3 (8) | 3.6 (3) | 1.6 (2) |
|  | Ground-to head | 3.8 (7) | 1.2 (1) | 4.1 (5) |
|  | Arm-to head | 3.8 (7) | 1.2 (1) | 2.4 (3) |
|  | Trunk-to head | 2.7 (5) | 2.4 (2) | 3.3 (4) |
|  | Foot-to head | 2.7 (5) | - | 6.5 (8) |
|  | Knee-to head | 2.2 (4) | 2.4 (2) | 1.6 (2) |
|  | Leg-to head | 1.1 (2) | 2.4 (2) | 1.6 (2) |
|  | Thigh-to head | - | 3.6 (3) | 3.3 (4) |
|  | Hand-to neck | 2.2 (4) | 2.4 (2) | 1.6 (2) |
|  | Elbow-to neck | 1.1 (2) | 1.2 (1) | - |
|  | Hip-to head | - | - | 1.6 (2) |
|  | Object-to head | - | 1.2 (1) | - |
|  | Elbow-to trunk | 0.5 (1) | - | - |
|  | Shoulder-to trunk | 0.5 (1) | - | - |
|  | Forearm-to upper back | 0.5 (1) | - | - |
|  | Forearm-to neck | 0.5 (1)- | - | - |
|  | Unclear | 2.2 (4) | - | 3.3 (4) |
|  | Total | 100 (184) | 100 (84) | 100 (123) |
| **Player action** | |  |  |  |
|  | Aerial duel | 45.1 (83) | 7.1 (6) | 22.8 (28) |
|  | Ball progression | 11.4 (21) | 17.9 (15) | 8.1 (10) |
|  | Other duels | 9.2 (17) | 19 (16) | 19.5 (24) |
|  | Attempt at goal | 6 (11) | 2.4 (2) | 4.9 (6) |
|  | Pressing | 5.4 (10) | 9.5 (8) | 4.1 (5) |
|  | Offering to receive | 4.9 (9) | 4.8 (4) | 4.1 (5) |
|  | Clearance | 4.3(8) | - | 4.9 (6) |
|  | Block | 3.8 (7) | 22.6 (19) | 17.1 (21) |
|  | Pass | 3.8 (7) | 3.6 (3) | 3.3 (4) |
|  | Tackle | 1.6 (3) | 6 (5) | 8.9 (11) |
|  | Pushing on | 1.1(2) | - | 0.8 (1) |
|  | Other action | 0.5 (1) | 7.1 (6) | - |
|  | Unclear | 2.7 (5) | - | 1.6 (2) |
|  | Total | 100 (184) | 100 (84) | 100 (123) |
| **Overhead kick** | |  |  |  |
|  | No | 100 (184) | 100) (84) | 82.1 (101) |
|  | Yes | - | - | 17.9 (22) |
|  | Total | 100 (184) | 100 (84) | 100 (123) |
| **Ball possession nature** | |  |  |  |
|  | Free ball | 61.4 (113) | 25 (21) | 39 (48) |
|  | Injured player | 15.8 (29) | 19 (16) | 17.9 (22) |
|  | Uninjured player | 12 (22) | 38.1 (32) | 29.3 (36) |
|  | Without ball | 10.9 (20) | 17.9 (15) | 13.8 (17) |
|  | Total | 100 (184) | 100 (84) | 100 (123) |
| **Team status** | |  |  |  |
|  | Attack | 42.9 (79) | 35.7 (30) | 39 (48) |
|  | Defence | 40.2 (74) | 52.4 (44) | 57.7 (71) |
|  | In contest | 16.8 (31) | 11.9 (10) | 3.3 (4) |
|  | Total | 100 (184) | 100 (84) | 123 |
| **Contested nature** | |  |  |  |
|  | Contested | 94 (173) | 83.3 (70) | 91.9 (113) |
|  | Uncontested | 6 (11) | 16.7 (14) | 8.1 (10) |
|  | Total | 100 (184) | 100 (84) | 100 (123) |
| **Protective body positioning** | |  |  |  |
|  | No protective action | 89.6 (155) | 98.6 (69) | 78.8 (89) |
|  | Protective action - turns away | 3.5 (6) | - | 9.7 (11) |
|  | Protective action – head | 1.7 (3) | - | 6.2 (7) |
|  | Protective action – arms | 4 (7) | 1.4 (1) | 3.5 (4) |
|  | Unclear | 1.2 (2) | - | 1.8 (2) |
|  | Total | 100 (173) | 100 (70) | 100 (113) |
| **Injured player intent during contested event** | |  |  |  |
|  | Compete to win the ball – head | 50.9 (88) | 10 (7) | 23 (26) |
|  | No competition | 16.8 (29) | 22.9 (16) | 23.9 (27) |
|  | The player has already had the ball | 14.5 (25) | 22.9 (16) | 15.9 (18) |
|  | Compete to win the ball – feet | 12.1 (21) | 30 (21) | 25.7 (29) |
|  | Compete to win the ball – other | 3.5 (6) | 2.9 (2) | 2.7 (3) |
|  | Goalkeeper competing for ball | 2.3 (4) | 70 (7) | 8 (9) |
|  | Interfere with opponent's action | - | 1.4 (1) | 0.9 (1) |
|  | Unclear | - | - | - |
|  | Total | 100 (173) | 100 (70) | 100 (113) |
| **Injured player awareness of other player** | |  |  |  |
|  | Yes | 83.8 (145) | 94.3 (66) | 93.8 (106) |
|  | No | 15 (26) | 5.7 (4) | 6.2 (7) |
|  | Unclear | 1.2 (2) | - | - |
|  | Total | 100 (173) | 100 (70) | 100 (113) |
| **Obstruction** | |  |  |  |
|  | No obstruction | 76.9 (133) | 68.6 (48) | 83.2 (94) |
|  | Yes - obstructed by opponent | 22 (38) | 30 (21) | 15.9 (18) |
|  | Yes - obstructed by teammate | 1.2 (2) | - | 0.9 (1) |
|  | Yes - obstructed by multiple players | - | 1.4 (1) | - |
|  | Unclear | - | - | - |
|  | Total | 100 (173) | 100 (70) | 100 (113) |
| **Interference** | |  |  |  |
|  | Interference – pushed off the ball | 56.1 (97) | 45.7 (32) | 45.1 (51) |
|  | No interference | 22.5 (39) | 35.7 (25) | 33.6 (38) |
|  | Interference – held off the ball | 12.1 (21) | 10 (7) | 10.6 (12) |
|  | Interference- teammate | 5.8 (10) | 2.9 (2) | - |
|  | Interference – opposition other | 2.9 (5) | 5.7 (4) | 10.6 (12) |
|  | Unclear | 0.6 (1) | - | - |
|  | Total | 100 (173) | 100 (70) | 100 (113) |
| **Red flags** | |  |  |  |
|  | No | 97.8 (180) | 100 (84) | 100) (123) |
|  | Yes | 2.2 (4) | - | - |
|  | Total | 100 (184) | 100 (84) | 100 (123) |
| **Yellow flags** | |  |  |  |
|  | No | 83.7 (154) | 90.5 (76) | 79.7 (98) |
|  | Yes | 16.3 (30) | 9.5 (8) | 20.3 (25) |
|  | Total | 100 (184) | 100 (84) | 100 (123) |
| **Outcomes** | |  |  |  |
| **The outcome of the injury** | |  |  |  |
|  | No medical evaluation | 78.2 (144) | 69 (58) | 82 (101) |
|  | Evaluated and returned to match | 20.7 (38) | 29.7 (25) | 14.7 (18) |
|  | Evaluated and substituted | 1.1 (2) | - | 3.3 (4) |
|  | Unclear | - | 1.2 (1) | - |
|  | Total | 100 (184) | 100 (84) | 100 (123) |
| **Referee decision** | |  |  |  |
|  | No sanction | 51.6 (95) | 51.2 (43) | 53.7 (66) |
|  | Foul + no card | 29.9 (55) | 27.4 (23) | 30.1 (37) |
|  | Foul + card | 11.9 (22) | 19.1 (16) | 11.4 (14) |
|  | Against foul | 6.5 (12) | 2.4 (2) | 4.6 (6) |
|  | Total | 100 (184) | 100 (84) | 100 (123) |
